# Poly[di-μ_2_-aqua-μ_5_-(pyridine-2,6-dicarboxyl­ato)-μ_3_-(pyridine-2,6-dicarboxyl­ato)-cobalt(II)disodium]

**DOI:** 10.1107/S1600536811048252

**Published:** 2011-11-19

**Authors:** Alexander N. Boyko, Irina A. Golenya, Yulia A. Izotova, Matti Haukka, Elena V. Prisyazhnaya

**Affiliations:** a Kiev National Taras Shevchenko University, Department of Chemistry, Volodymyrska Street 64, 01601 Kiev, Ukraine; b Department of Chemistry, St Petersburg State University, Universitetsky Pr. 26, 198504 Stary Petergof, Russian Federation; c University of Joensuu, Department of Chemistry, PO Box 111, FI-80101 Joensuu, Finland; d Kyiv National University of Construction and Architecture, Department of Chemistry, Povitroflotsky Avenue 31, 03680 Kiev, Ukraine

## Abstract

In the title compound, [CoNa_2_(C_7_H_3_NO_4_)_2_(H_2_O)_2_]_*n*_, the Co^II^ atom is coordinated by two pyridine N atoms and four carboxyl­ate O atoms from two doubly deprotonated pyridine-2,6-dicarboxyl­ate ligands in a distorted octa­hedral geometry. One Na^+^ cation is coordinated by three carboxyl­ate O atoms and two water mol­ecules and the other is coordinated by five carboxyl­ate O atoms and two water mol­ecules in an irregular geometry. The bis­(pyridine-2,6-dicarboxyl­ato)cobalt complex units are connected by Na^+^ cations and bridging water mol­ecules into a three-dimensional coordination network. O—H⋯O hydrogen bonds are formed between the water mol­ecules and the carboxyl­ate O atoms.

## Related literature

For hydrolytic decomposition of hydroxamate ligands upon complex formation, see: Dobosz *et al.* (1999 ▶); Świątek-Kozłowska *et al.* (2000 ▶). For related structures, see: Fritsky *et al.* (2001 ▶); Krämer & Fritsky (2000 ▶); Mokhir *et al.* (2002 ▶); Moroz *et al.* (2010 ▶); Sachse *et al.* (2008 ▶); Sliva *et al.* (1997 ▶); Wörl *et al.* (2005*a* ▶,*b* ▶). For the preparation of the ligand, see: Świątek-Kozłowska *et al.* (2002 ▶). 
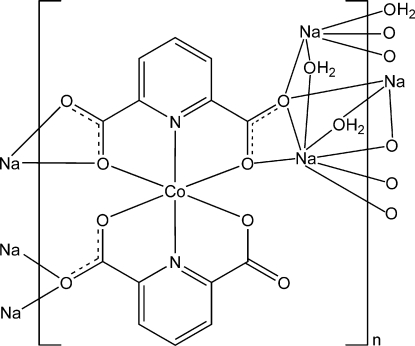


## Experimental

### 

#### Crystal data

[CoNa_2_(C_7_H_3_NO_4_)_2_(H_2_O)_2_]
*M* *_r_* = 471.15
Orthorhombic, 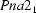

*a* = 7.9540 (3) Å
*b* = 13.2187 (3) Å
*c* = 15.1475 (3) Å
*V* = 1592.63 (8) Å^3^
*Z* = 4
Mo *K*α radiation
μ = 1.20 mm^−1^
*T* = 120 K
0.17 × 0.10 × 0.06 mm

#### Data collection

Nonius KappaCCD diffractometer
Absorption correction: multi-scan (*DENZO*/*SCALEPACK*; Otwinowski & Minor, 1997 ▶) *T*_min_ = 0.826, *T*_max_ = 0.931
22979 measured reflections
3640 independent reflections
3388 reflections with *I* > 2σ(*I*)
*R*_int_ = 0.037

#### Refinement

*R*[*F* ^2^ > 2σ(*F* ^2^)] = 0.023
*wR*(*F* ^2^) = 0.054
*S* = 1.07
3640 reflections
264 parameters
1 restraint
H-atom parameters constrained
Δρ_max_ = 0.30 e Å^−3^
Δρ_min_ = −0.32 e Å^−3^
Absolute structure: Flack (1983 ▶), 1749 Friedel pairs
Flack parameter: 0.017 (10)

### 

Data collection: *COLLECT* (Nonius, 1998 ▶); cell refinement: *DENZO*/*SCALEPACK* (Otwinowski & Minor, 1997 ▶); data reduction: *DENZO*/*SCALEPACK*; program(s) used to solve structure: *SIR2004* (Burla *et al.*, 2005 ▶); program(s) used to refine structure: *SHELXL97* (Sheldrick, 2008 ▶); molecular graphics: *DIAMOND* (Brandenburg, 1999 ▶); software used to prepare material for publication: *SHELXL97*.

## Supplementary Material

Crystal structure: contains datablock(s) I, global. DOI: 10.1107/S1600536811048252/hy2486sup1.cif

Structure factors: contains datablock(s) I. DOI: 10.1107/S1600536811048252/hy2486Isup2.hkl

Additional supplementary materials:  crystallographic information; 3D view; checkCIF report

## Figures and Tables

**Table 1:** Hydrogen-bond geometry (Å, °)

*D*—H⋯*A*	*D*—H	H⋯*A*	*D*⋯*A*	*D*—H⋯*A*
O9—H9*A*⋯O7^i^	0.84	1.94	2.742 (2)	159
O9—H9*B*⋯O4^ii^	0.85	1.90	2.717 (2)	162
O10—H10*A*⋯O3	0.86	1.88	2.725 (2)	166
O10—H10*B*⋯O4^iii^	0.91	2.12	2.993 (2)	159

Symmetry codes: (i) 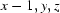; (ii) 
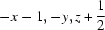
; (iii) 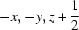.

**Fig. 1.**
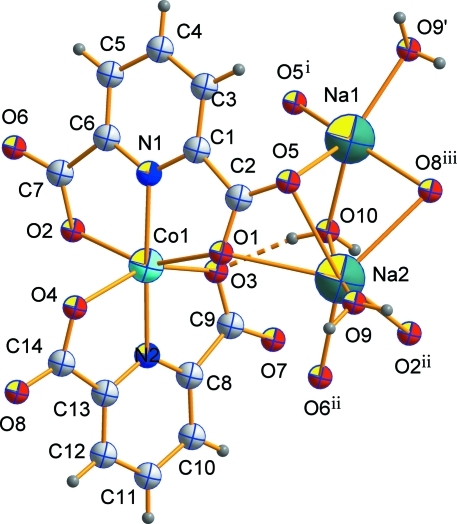
The asymmetric unit of the title compound. Displacement ellipsoinds are shown at the 50% probability level. Hydrogen bonds are indicated by dashed lines. [Symmetry codes: (i) 1/2+x, 1/2-y, z; (ii) -x, -y, 1/2+z; (iii) -1/2-x, 1/2+y, 1/2+z.]

**Fig. 2.**
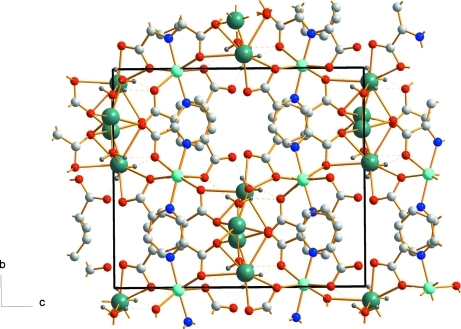
A view of the crystal packing for the title compound.

## supplementary crystallographic information

### Comment

Hydroxamic acids are widely used in synthesis of polynuclear compounds and coordination polymers. However, when the synthesis is conducted in alkaline conditions, hydrolytic decomposition of the hydroxamate function resulting in the formation of carboxylic groups sometimes occurs (Dobosz *et al.*, 1999; Świątek-Kozłowska *et al.*, 2000). Herein we report the crystal structure of the title compound obtained as a result of hydrolytic decomposition of pyridine-2,6-dihydroxamic acid in the course of formation of the anionic complex with cobalt(II) in the presence of sodium hydroxide.

In the title compound, the Co^II^ atom is coordinated by two pyridine N atoms and four carboxylate O atoms from two doubly deprotonated pyridine-2,6-dicarboxylate ligands in a distorted octahedral geometry (Fig. 1). The Co—O bond lengths are in a range of 2.1235 (17)–2.2065 (14) Å, which clearly indicates that the central ion is in bivalent state (Sliva *et al.*, 1997). The Na ions are coordinated by O atoms of pyridine-2,6-dicarboxylate ligands and two water molecules in irregular geometries. Na1 atom is in a strongly distorted square-pyramidal environment, while Na2 atom exhibits a coordination number 7 and its geometry approaches to a distorted pentagonal-bipyramidal. The Na—O bond lengths lie in a range of 2.2756 (11)–2.7557 (17) Å, which is normal for sodium ions (Mokhir *et al.*, 2002; Świątek-Kozłowska *et al.*, 2000). The C9—O3 and C9—O7 bond lengths [1.284 (2) and 1.240 (2) Å] are typical for a monodentately coordinated carboxylate (Wörl *et al.*, 2005*a*,*b*). The C—N and C—C bond lengths in the pyridine rings are normal for 2-substituted pyridine derivatives (Fritsky *et al.*, 2001; Krämer & Fritsky, 2000; Moroz *et al.*, 2010; Sachse *et al.*, 2008).

In the crystal packing (Fig. 2), the bis(pyridine-2,6-dicarboxylato)cobalt(II) complex units are connected by the sodium ions and water molecules into a three-dimensional coordination network. The two water molecules bridge the sodium ions and form intermolecular hydrogen bonds (Table 1).

### Experimental

Cobalt(II) perchlorate hexahydrate (0.0365 g, 0.1 mmol) was dissolved in methanol (5 ml) and mixed with a solution of pyridine-2,6-dihydroxamic acid (0.0197 g, 0.1 mmol) synthesized according to Świątek-Kozłowska *et al.* (2002) in H_2_O, and then to the obtained mixture solution sodium hydroxide (0.1 M, 1 ml) was added. The mixture was stirred for 30 min and filtered. The insoluble material was dissolved in DMSO (3 ml) and set aside for crystallization by slow diffusion of isopropanol vapours into the formed solution. The greenish yellow crystals formed in 5–7 days were filtered off, washed with isopropanol and dried (yield: 78%).

### Refinement

The water H atoms were located from a difference Fourier map and constrained to ride on their parent atom, with *U*_iso_(H) = 1.5*U*_eq_(O). Other H atoms were positioned geometrically and also constrained to ride on their parent atoms, with C—H = 0.95 Å and *U*_iso_(H) = 1.2*U*_eq_(C). The highest peak is located 1.59 Å from atom O3 and the deepest hole is located 0.66 Å from atom Co1.

### Crystal data

**Table:**

[CoNa_2_(C_7_H_3_NO_4_)_2_(H_2_O)_2_]	*F*(000) = 948
*M**_r_* = 471.15	*D*_x_ = 1.965 Mg m^−^^3^
Orthorhombic, *P**n**a*2_1_	Mo *K*α radiation, λ = 0.71073 Å
Hall symbol: P 2c -2n	Cell parameters from 14413 reflections
*a* = 7.9540 (3) Å	θ = 1.0–27.5°
*b* = 13.2187 (3) Å	µ = 1.20 mm^−^^1^
*c* = 15.1475 (3) Å	*T* = 120 K
*V* = 1592.63 (8) Å^3^	Block, green-yellow
*Z* = 4	0.17 × 0.10 × 0.06 mm

### Data collection

**Table:**

Nonius KappaCCD diffractometer	3640 independent reflections
Radiation source: fine-focus sealed tube	3388 reflections with *I* > 2σ(*I*)
horizontally mounted graphite crystal	*R*_int_ = 0.037
Detector resolution: 9 pixels mm^-1^	θ_max_ = 27.5°, θ_min_ = 3.1°
φ and ω scans with κ offset	*h* = −10→10
Absorption correction: multi-scan (*DENZO*/*SCALEPACK*; Otwinowski & Minor, 1997)	*k* = −17→15
*T*_min_ = 0.826, *T*_max_ = 0.931	*l* = −19→19
22979 measured reflections	

### Refinement

**Table:**

Refinement on *F*^2^	Secondary atom site location: difference Fourier map
Least-squares matrix: full	Hydrogen site location: mixed
*R*[*F*^2^ > 2σ(*F*^2^)] = 0.023	H-atom parameters constrained
*wR*(*F*^2^) = 0.054	*w* = 1/[σ^2^(*F*_o_^2^) + (0.0247*P*)^2^ + 0.349*P*] where *P* = (*F*_o_^2^ + 2*F*_c_^2^)/3
*S* = 1.07	(Δ/σ)_max_ < 0.001
3640 reflections	Δρ_max_ = 0.30 e Å^−^^3^
264 parameters	Δρ_min_ = −0.32 e Å^−^^3^
1 restraint	Absolute structure: Flack (1983), 1749 Friedel pairs
Primary atom site location: structure-invariant direct methods	Flack parameter: 0.017 (10)

### Fractional atomic coordinates and isotropic or equivalent isotropic displacement parameters (Å^2^)

**Table:**

	*x*	*y*	*z*	*U*_iso_*/*U*_eq_	
Co1	−0.05451 (3)	−0.010675 (17)	0.25557 (2)	0.01076 (7)	
Na1	−0.07221 (11)	0.28100 (6)	0.49042 (5)	0.01966 (19)	
Na2	−0.26548 (10)	0.06445 (7)	0.52208 (6)	0.0212 (2)	
O1	−0.24152 (18)	0.05370 (10)	0.34732 (9)	0.0146 (3)	
O2	0.1115 (2)	0.00400 (10)	0.14651 (11)	0.0163 (4)	
O3	0.1244 (2)	−0.00136 (10)	0.36280 (10)	0.0141 (3)	
O4	−0.21550 (19)	−0.09844 (10)	0.16519 (10)	0.0158 (3)	
O5	−0.30854 (18)	0.20122 (10)	0.41058 (9)	0.0162 (3)	
O6	0.18157 (18)	0.10750 (11)	0.03583 (9)	0.0183 (3)	
O7	0.23230 (19)	−0.09423 (11)	0.47351 (9)	0.0180 (3)	
O8	−0.2541 (2)	−0.25677 (11)	0.11508 (9)	0.0188 (3)	
O9	−0.55522 (17)	0.05090 (11)	0.53885 (10)	0.0160 (3)	
H9A	−0.5987	0.0012	0.5123	0.024*	
H9B	−0.6164	0.0561	0.5846	0.024*	
O10	0.01645 (19)	0.11758 (10)	0.49835 (10)	0.0164 (3)	
H10A	0.0629	0.0876	0.4541	0.025*	
H10B	0.0745	0.0949	0.5461	0.025*	
N1	−0.0691 (2)	0.13931 (12)	0.22358 (10)	0.0109 (3)	
N2	−0.0236 (2)	−0.15690 (12)	0.29271 (11)	0.0112 (3)	
C1	−0.1490 (2)	0.20301 (14)	0.27769 (12)	0.0120 (4)	
C2	−0.2419 (2)	0.14940 (15)	0.35081 (13)	0.0126 (4)	
C3	−0.1404 (2)	0.30715 (14)	0.26432 (15)	0.0158 (4)	
H3	−0.1946	0.3527	0.3036	0.019*	
C4	−0.0503 (3)	0.34270 (15)	0.19181 (14)	0.0153 (4)	
H4	−0.0429	0.4134	0.1812	0.018*	
C5	0.0285 (3)	0.27576 (16)	0.13529 (14)	0.0151 (4)	
H5	0.0879	0.2992	0.0849	0.018*	
C6	0.0182 (3)	0.17334 (15)	0.15435 (13)	0.0121 (4)	
C7	0.1110 (3)	0.09008 (15)	0.10677 (13)	0.0136 (4)	
C8	0.0704 (3)	−0.17728 (16)	0.36365 (13)	0.0121 (4)	
C9	0.1491 (3)	−0.08438 (15)	0.40480 (13)	0.0136 (4)	
C10	0.0974 (3)	−0.27604 (15)	0.39155 (14)	0.0153 (4)	
H10	0.1621	−0.2900	0.4429	0.018*	
C11	0.0270 (3)	−0.35394 (15)	0.34216 (13)	0.0149 (4)	
H11	0.0446	−0.4222	0.3594	0.018*	
C12	−0.0685 (2)	−0.33259 (14)	0.26795 (15)	0.0148 (4)	
H12	−0.1161	−0.3854	0.2335	0.018*	
C13	−0.0928 (2)	−0.23155 (14)	0.24533 (14)	0.0120 (4)	
C14	−0.1961 (3)	−0.19475 (15)	0.16881 (13)	0.0131 (4)	

### Atomic displacement parameters (Å^2^)

**Table:**

	*U*^11^	*U*^22^	*U*^33^	*U*^12^	*U*^13^	*U*^23^
Co1	0.01387 (12)	0.00901 (11)	0.00941 (12)	0.00019 (9)	0.00036 (12)	0.00034 (13)
Na1	0.0260 (5)	0.0149 (4)	0.0181 (4)	−0.0034 (3)	0.0049 (3)	−0.0026 (3)
Na2	0.0140 (4)	0.0243 (5)	0.0251 (4)	0.0016 (3)	0.0007 (4)	0.0109 (4)
O1	0.0164 (8)	0.0128 (7)	0.0147 (7)	−0.0013 (6)	0.0015 (6)	0.0004 (6)
O2	0.0214 (9)	0.0125 (8)	0.0151 (9)	0.0016 (6)	0.0058 (7)	0.0001 (6)
O3	0.0164 (9)	0.0130 (8)	0.0130 (9)	−0.0006 (6)	−0.0009 (7)	−0.0006 (5)
O4	0.0194 (8)	0.0142 (7)	0.0137 (7)	−0.0007 (6)	−0.0030 (6)	0.0014 (6)
O5	0.0180 (8)	0.0181 (7)	0.0124 (7)	0.0024 (6)	0.0024 (6)	−0.0020 (6)
O6	0.0217 (8)	0.0203 (8)	0.0130 (7)	−0.0022 (6)	0.0057 (6)	0.0004 (6)
O7	0.0194 (8)	0.0201 (8)	0.0145 (8)	0.0017 (6)	−0.0067 (6)	−0.0003 (6)
O8	0.0254 (8)	0.0168 (8)	0.0141 (7)	−0.0049 (6)	−0.0050 (6)	−0.0016 (6)
O9	0.0162 (7)	0.0137 (7)	0.0181 (7)	−0.0016 (6)	0.0037 (6)	−0.0029 (6)
O10	0.0184 (8)	0.0186 (7)	0.0120 (7)	0.0018 (6)	−0.0001 (6)	−0.0010 (6)
N1	0.0121 (9)	0.0111 (8)	0.0095 (7)	0.0003 (7)	−0.0010 (6)	−0.0010 (6)
N2	0.0117 (8)	0.0112 (8)	0.0108 (8)	0.0002 (7)	0.0008 (6)	0.0001 (6)
C1	0.0104 (9)	0.0138 (9)	0.0118 (10)	0.0012 (8)	−0.0021 (7)	−0.0012 (7)
C2	0.0118 (10)	0.0154 (10)	0.0106 (9)	0.0019 (8)	−0.0016 (8)	−0.0011 (8)
C3	0.0164 (9)	0.0128 (8)	0.0180 (11)	0.0029 (7)	−0.0012 (9)	−0.0014 (9)
C4	0.0182 (11)	0.0090 (9)	0.0185 (10)	0.0005 (8)	−0.0042 (8)	0.0049 (8)
C5	0.0162 (11)	0.0169 (10)	0.0123 (10)	−0.0014 (8)	−0.0024 (8)	0.0045 (8)
C6	0.0115 (10)	0.0136 (10)	0.0114 (10)	−0.0008 (8)	−0.0011 (8)	−0.0006 (8)
C7	0.0121 (10)	0.0163 (10)	0.0125 (10)	−0.0029 (8)	−0.0009 (8)	−0.0016 (8)
C8	0.0105 (11)	0.0145 (10)	0.0111 (10)	0.0005 (7)	0.0029 (7)	0.0008 (8)
C9	0.0112 (10)	0.0160 (10)	0.0137 (10)	0.0021 (8)	0.0023 (8)	0.0001 (8)
C10	0.0152 (10)	0.0158 (11)	0.0150 (10)	0.0030 (8)	0.0025 (8)	0.0040 (8)
C11	0.0165 (11)	0.0116 (10)	0.0167 (10)	0.0039 (8)	0.0025 (8)	0.0040 (8)
C12	0.0167 (10)	0.0115 (8)	0.0163 (11)	−0.0002 (7)	0.0064 (8)	−0.0033 (8)
C13	0.0124 (9)	0.0128 (8)	0.0108 (10)	0.0001 (7)	0.0029 (8)	−0.0025 (8)
C14	0.0134 (10)	0.0140 (9)	0.0119 (9)	−0.0006 (8)	0.0038 (8)	0.0036 (8)

### Geometric parameters (Å, °)

**Table:**

Co1—N2	2.0281 (16)	O9—H9A	0.8440
Co1—N1	2.0443 (17)	O9—H9B	0.8492
Co1—O2	2.1235 (17)	O10—H10A	0.8618
Co1—O3	2.1631 (17)	O10—H10B	0.9091
Co1—O4	2.2044 (15)	N1—C6	1.336 (3)
Co1—O1	2.2065 (14)	N1—C1	1.336 (2)
Na1—O10	2.2757 (16)	N2—C8	1.337 (3)
Na1—O9^i^	2.3439 (16)	N2—C13	1.339 (2)
Na1—O8^ii^	2.3924 (16)	C1—C3	1.393 (3)
Na1—O5^i^	2.4324 (16)	C1—C2	1.508 (3)
Na1—O5	2.4715 (16)	C3—C4	1.393 (3)
Na2—O9	2.3254 (16)	C3—H3	0.9500
Na2—O10	2.3772 (17)	C4—C5	1.382 (3)
Na2—O6^iii^	2.3780 (17)	C4—H4	0.9500
Na2—O2^iii^	2.4232 (18)	C5—C6	1.387 (3)
Na2—O5	2.4978 (16)	C5—H5	0.9500
Na2—O1	2.6579 (17)	C6—C7	1.508 (3)
Na2—O8^ii^	2.7557 (17)	C8—C10	1.389 (3)
O1—C2	1.266 (2)	C8—C9	1.513 (3)
O2—C7	1.287 (2)	C10—C11	1.391 (3)
O3—C9	1.284 (2)	C10—H10	0.9500
O4—C14	1.284 (2)	C11—C12	1.386 (3)
O5—C2	1.253 (2)	C11—H11	0.9500
O6—C7	1.234 (2)	C12—C13	1.392 (3)
O7—C9	1.240 (2)	C12—H12	0.9500
O8—C14	1.244 (2)	C13—C14	1.502 (3)
			
N2—Co1—N1	175.53 (7)	C14—O8—Na2^vi^	151.48 (14)
N2—Co1—O2	103.15 (6)	Na1^vi^—O8—Na2^vi^	78.94 (5)
N1—Co1—O2	76.24 (6)	Na2—O9—Na1^v^	87.13 (5)
N2—Co1—O3	76.47 (6)	Na2—O9—H9A	114.5
N1—Co1—O3	99.21 (6)	Na1^v^—O9—H9A	124.4
O2—Co1—O3	99.78 (5)	Na2—O9—H9B	130.6
N2—Co1—O4	75.00 (6)	Na1^v^—O9—H9B	98.4
N1—Co1—O4	109.28 (6)	H9A—O9—H9B	102.5
O2—Co1—O4	85.76 (6)	Na1—O10—Na2	89.78 (6)
O3—Co1—O4	151.46 (5)	Na1—O10—H10A	121.9
N2—Co1—O1	105.94 (6)	Na2—O10—H10A	112.7
N1—Co1—O1	74.76 (6)	Na1—O10—H10B	120.8
O2—Co1—O1	150.91 (5)	Na2—O10—H10B	105.1
O3—Co1—O1	87.05 (6)	H10A—O10—H10B	104.4
O4—Co1—O1	101.68 (5)	C6—N1—C1	121.13 (17)
O10—Na1—O9^i^	149.94 (7)	C6—N1—Co1	118.90 (13)
O10—Na1—O8^ii^	86.51 (6)	C1—N1—Co1	119.52 (13)
O9^i^—Na1—O8^ii^	89.09 (5)	C8—N2—C13	120.84 (17)
O10—Na1—O5^i^	81.42 (6)	C8—N2—Co1	118.88 (14)
O9^i^—Na1—O5^i^	90.82 (5)	C13—N2—Co1	120.26 (13)
O8^ii^—Na1—O5^i^	155.57 (6)	N1—C1—C3	120.67 (17)
O10—Na1—O5	81.75 (5)	N1—C1—C2	112.81 (17)
O9^i^—Na1—O5	126.98 (6)	C3—C1—C2	126.53 (17)
O8^ii^—Na1—O5	81.84 (5)	O5—C2—O1	125.27 (18)
O5^i^—Na1—O5	116.98 (6)	O5—C2—C1	118.75 (17)
O9—Na2—O10	166.94 (6)	O1—C2—C1	115.96 (17)
O9—Na2—O6^iii^	101.24 (6)	C1—C3—C4	118.24 (18)
O10—Na2—O6^iii^	91.77 (5)	C1—C3—H3	120.9
O9—Na2—O2^iii^	112.79 (7)	C4—C3—H3	120.9
O10—Na2—O2^iii^	75.58 (6)	C5—C4—C3	120.39 (19)
O6^iii^—Na2—O2^iii^	55.47 (5)	C5—C4—H4	119.8
O9—Na2—O5	89.64 (6)	C3—C4—H4	119.8
O10—Na2—O5	79.24 (5)	C4—C5—C6	118.01 (19)
O6^iii^—Na2—O5	142.14 (6)	C4—C5—H5	121.0
O2^iii^—Na2—O5	149.95 (6)	C6—C5—H5	121.0
O9—Na2—O1	100.12 (6)	N1—C6—C5	121.52 (19)
O10—Na2—O1	78.32 (5)	N1—C6—C7	112.57 (17)
O6^iii^—Na2—O1	90.91 (5)	C5—C6—C7	125.74 (19)
O2^iii^—Na2—O1	135.93 (6)	O6—C7—O2	124.80 (19)
O5—Na2—O1	51.32 (4)	O6—C7—C6	120.17 (18)
O9—Na2—O8^ii^	93.80 (6)	O2—C7—C6	115.02 (17)
O10—Na2—O8^ii^	76.74 (5)	N2—C8—C10	121.37 (19)
O6^iii^—Na2—O8^ii^	139.38 (6)	N2—C8—C9	113.53 (17)
O2^iii^—Na2—O8^ii^	83.92 (6)	C10—C8—C9	125.01 (19)
O5—Na2—O8^ii^	74.50 (5)	O7—C9—O3	125.97 (19)
O1—Na2—O8^ii^	123.43 (5)	O7—C9—C8	118.77 (18)
C7^iii^—Na2—O8^ii^	112.28 (6)	O3—C9—C8	115.24 (17)
C2—O1—Co1	114.40 (12)	C8—C10—C11	118.04 (19)
C2—O1—Na2	84.54 (11)	C8—C10—H10	121.0
Co1—O1—Na2	134.13 (6)	C11—C10—H10	121.0
C7—O2—Co1	116.28 (13)	C12—C11—C10	120.42 (18)
C7—O2—Na2^iv^	88.16 (12)	C12—C11—H11	119.8
Co1—O2—Na2^iv^	152.28 (7)	C10—C11—H11	119.8
C9—O3—Co1	115.10 (13)	C11—C12—C13	118.10 (19)
C14—O4—Co1	115.19 (13)	C11—C12—H12	121.0
C2—O5—Na1^v^	140.98 (13)	C13—C12—H12	121.0
C2—O5—Na1	105.37 (12)	N2—C13—C12	121.21 (18)
Na1^v^—O5—Na1	111.79 (6)	N2—C13—C14	113.58 (16)
C2—O5—Na2	92.00 (12)	C12—C13—C14	125.21 (18)
Na1^v^—O5—Na2	81.48 (5)	O8—C14—O4	125.53 (19)
Na1—O5—Na2	82.74 (5)	O8—C14—C13	119.63 (17)
C7—O6—Na2^iv^	91.47 (12)	O4—C14—C13	114.84 (17)
C14—O8—Na1^vi^	126.37 (13)		

Symmetry codes: (i) *x*+1/2, −*y*+1/2, *z*; (ii) −*x*−1/2, *y*+1/2, *z*+1/2; (iii) −*x*, −*y*, *z*+1/2; (iv) −*x*, −*y*, *z*−1/2; (v) *x*−1/2, −*y*+1/2, *z*; (vi) −*x*−1/2, *y*−1/2, *z*−1/2.

### Hydrogen-bond geometry (Å, °)

**Table:**

*D*—H···*A*	*D*—H	H···*A*	*D*···*A*	*D*—H···*A*
O9—H9A···O7^vii^	0.84	1.94	2.742 (2)	159
O9—H9B···O4^viii^	0.85	1.90	2.717 (2)	162
O10—H10A···O3	0.86	1.88	2.725 (2)	166
O10—H10B···O4^iii^	0.91	2.12	2.993 (2)	159

Symmetry codes: (vii) *x*−1, *y*, *z*; (viii) −*x*−1, −*y*, *z*+1/2; (iii) −*x*, −*y*, *z*+1/2.
